# The Future Outlook

**Published:** 1919-09-06

**Authors:** 


### GRADUATE MEDICAL INSTRUCTION.

### The Future Outlook.

The end of the war has brought home to us the need of graduate instruction. We are also faced with the fact that there is a golden opportunity to capture the teaching that used to be given in Germany, and more especially in Austria. We may also feel assured that, if we are not in a position to give such instruction, those wishing to get it will return to those countries in a few years' time. The popularity of Vienna for English-speaking graduates was due to two factors—that they had there devised and put on the market a system which satisfied the needs of those who wanted graduate teaching, and that no such systematic teaching was obtainable in any part of the British Isles.

In Vienna various teachers volunteered to take classes in a given subject. Such classes were for a definite number of attendances, each of a definite time, over a definite period. The number of students admitted to a course was limited. As soon as the requisite number had put down their names the course began. Once it had begun no one else was admitted to the course, so that it was not necessary to go over the same point twice. By putting his name down for several courses a student could fill up part of his time. The rest of the time was spent in attending clinics, generally those of the men whose course he was attending or had attended. In addition there were lectures of general interest organised in the evening or late afternoon, so as not to interfere with the other work. The advantages of this method were great. The student could fill in his time at his own subject; he could get full advantage from a clinic, because he saw there work which he had heard discussed in a class. In this way it was not necessary for the physician holding the clinic to stop and explain things at the time, and so he could get on with the work. But the essence of the system was the limited class. The teacher knew how many men he could instruct, and more than that number were not admitted. Thus to a theoretical course of some branch of medicine or to a class on the surgical anatomy of some special subject as many as twenty would be admitted, to a demonstration of selected skin or surgical cases about twelve, while to a course on the physical examination of the chest, on the routine examination of the nose, throat, and ear, or on retinoscopy, about six would be admitted. To an operative surgery course would be admitted such a number for whom material could be provided and over whom personal supervision could be exercised. The fees for each course varied in correspondence—the smaller the number of men the larger the fee; but when a man had decided that he needed to take a course for which the fee was high he was seldom disappointed with the course, and thought at the end that it was worth it.

In Britain a man could go and attend any clinic, see cases, watch operations. There were occasional single lectures on general subjects. But for a man who wished to relearn some part of general medicine or to study more fully some speciality there was no systematic training. In a month or so a man cannot revise the whole of medicine; he attends graduate courses to rub up some little bit: hearts one year, chests the next, nerves a third; or he goes to begin to study some special branch; having had a grounding in general surgery, he wants to branch out in urology, or nose and throat, ear, or eye work. For all these we did not cater. We must see that in the future we do so. In Vienna the bulk of men were Americans, knowing what they wanted, and for this they were ready to pay. To the teacher who gave them what they wanted they flocked; to one who did not do so they did not go. After them came British colonials from Canada, Australia, and New Zealand, in character like the Americans, but generally of a higher standard of medical proficiency; then a few Englishmen and Scotsmen. If there were good teaching in our own countries these last would increase in number, and all the others would prefer to come to us. In the past in these isles we have claimed that we give the finest undergraduate medical training in the world, and turn out the best practitioner. We have feared that by doing graduate work we might interfere with our undergraduate training. This fear is a real one, but it must be faced. It must be possible to organise the teaching so that graduate instruction may be given without interfering with undergraduate work. Such is the ideal of the newly formed Graduate Medical Association. We hope it will prosper and wish it every success.

### Some Horrors of the Past.

For the reasons indicated not only Viennese but German teachers were favoured by graduates who wished to equip themselves fully to take a high place in their profession. But the resort to Vienna and Germany was not free from grave evils, owing to the inhumanities with which graduates were liable to be made acquainted. Space is precious to-day, and we confine ourselves to the publication of the following experience, told to him, substantially, by an attendant at these foreign clinics:

The place was a clinic in Berlin devoted to experimental and graduate teaching work in obstetrics and gynæcology. The work in hand was that on the drug now known as pituitrin as applied to the pregnant uterus. At the time neither the best preparation of the drug, nor its correct dosage, nor its exact mode of action was understood. Experiments were made in a manner remarkable for heartlessness. A number of women were injected with varying doses, regardless of over-dose and after-effect. The whole was done much in the same way that we inject rabbits in an experimental laboratory, and although, undoubtedly, valuable observations may have been made, they were obtained at the price of several human lives. Knowing the action of the drug, one can appreciate also that many of the patients who did not die must have been subjected to the acutest unnecessary pain by this rough-and-ready proceeding. All the effects of premature, excessive, and permanent contraction of the uterus would naturally arise.

C

**Graduate Medical Instruction—(*continued*).**

The Future.

The war has taught us that the mentality of these nations, whether scientists or soldiers, is such as to make them indifferent to and unmoved by the infliction of pain and terrible suffering on men, women, children, and presumably on animals too. It is clear, therefore, that the resort to these foreign schools in future for special graduate instruction should, and we hope will, terminate. The civilised world will expect that our British and American men of science, and all clean-minded and humane members of the human family, will seek their medical and scientific knowledge and practice elsewhere than in Germany and Austria. Next week we hope to demonstrate where and how the highest instruction and knowledge can be procured under the best conditions, free from the abominations and cruelties which we have felt it our duty to record. The Americans and the British have been and are building up great centres of instruction for graduates of all ages, and instruction in all fields of scientific and medical development.

